# Supplementary material for: An artificial intelligence approach for investigating multifactorial pain-related features of endometriosis
Source: PLoS One. 2024 Feb 21;19(2):e0297998. doi: 10.1371/journal.pone.0297998 (PMC10881015; doi:10.1371/journal.pone.0297998)
Supplement: S1 Table — These questions were used to determine the presence of specific clinical features in participants of the ENDO study. (Buck Louis GM, Hediger ML, Peterson CM, et al. Incidence of endometriosis by study population and diagnostic method: the ENDO study. Fertil Steril. 2011;96(2):360–365). (PDF) [file pone.0297998.s001.pdf]

S1 Table. Clinical features with corresponding survey questions.

Clinical Feature	Survey Questions
Abdominal pain for at least 12 weeks	In the past 12 months, have you had at least 12 weeks when you have had abdominal pain?
Chronic pelvic pain	Do you have pelvic pain that has been present longer than six months that affects your normal function?
Dyschezia	In the last six months, have you experienced pain with bowel elimination? (VAS 11-point scale; any pain > 1 counted as positive response)
Dysmenorrhea	In the last six months, have you experienced menstrual cramps rated 8 or more on VAS? Have you experienced painful menstrual cramps for more than 6 months that inhibit your normal activities and are not relieved with over-the-counter pain medications?
Dyspareunia	In the last six months, have you experienced vaginal pain with intercourse, deep pain with intercourse, burning vaginal pain after intercourse, or pelvic pain lasting hours or days after intercourse? (VAS 11-point scale; any pain > 1 counted as positive response)
Dysuria	In the last six months, have you experienced pain when bladder is full or pain with urination? (VAS 11-point scale; any pain > 1 counted as positive response)
Muscle or joint pain	In the last six months, have you experienced muscle or joint pain? (VAS 11-point scale; any pain > 1 counted as positive response)
Ovulation pain	In the last six months, have you experienced pain at ovulation? (VAS 11-point scale; any pain > 1 counted as positive response)
Subfertility	Have you ever tried to get pregnant for 6 months or more? Did you ever seek treatment for infertility?

These questions were used to determine the presence of specific clinical features in participants of the ENDO study. (Buck Louis GM, Hediger ML, Peterson CM, et al. Incidence of endometriosis by study population and diagnostic method: the ENDO study. Fertil Steril. 2011;96(2):360-365.)
