# Supplementary material for: An artificial intelligence approach for investigating multifactorial pain-related features of endometriosis
Source: PLoS One. 2024 Feb 21;19(2):e0297998. doi: 10.1371/journal.pone.0297998 (PMC10881015; doi:10.1371/journal.pone.0297998)
Supplement: S2 Table — Clusters were grouped as described in the methods. Location lists the anatomical sites included in each cluster. Asterisks in the Custer ID column denote clusters with N<25 participants. (PDF) [file pone.0297998.s002.pdf]

S2 Table. Clustered pain map areas.

Cluster ID	Location
1*	Front of neck
2	Urethra
3	Pelvis, groin, and sacrum
4	Hips, gluteus, lumbar, upper thigh, vulva, perineum
5	Right hypochondrium
6	Right and middle epigastrium
7*	Breasts
8	Sternum
9*	Left epigastrium
10*	Left hypochondrium
11	Subscapular
12*	Front of wrists
13*	Right clavicle
14	Lower limbs, upper back, chest, inner thighs
15*	Right forearm, back of wrists

Clusters were grouped as described in the methods. Location lists the anatomical sites included in each cluster. Asterisks in the Custer ID column denote clusters with N<25 participants.
