# Supplementary material for: An artificial intelligence approach for investigating multifactorial pain-related features of endometriosis
Source: PLoS One. 2024 Feb 21;19(2):e0297998. doi: 10.1371/journal.pone.0297998 (PMC10881015; doi:10.1371/journal.pone.0297998)
Supplement: S3 Table — This table presents a univariate analysis done with traditional statistical techniques. Significant differences were assessed using Fisher’s Exact Test. P-value adjusted for the false discovery rate. Pairwise comparisons for any significant associations were evaluated with Boschloo’s Exact Test. a,b,c,d,e,fSignificantly different pairwise values p-value < 0.05. (PDF) [file pone.0297998.s003.pdf]

S3 Table. Characteristics of the study sample by postoperative diagnosis.

Symptom	Endometriosis (n=190)	Uterine Fibroids (n=59)	Benign Ovarian Cysts (n=52)	Other Gynecological Condition (n=50)	Normal Pelvis (n=122)	Adjusted P-value
Chronic pelvic pain	146 (76.8%)[acd]	24 (40.7%)[a]	32 (61.5%)	27 (54.0%)[c]	58 (47.5%)[d]	< 0.001
Subfertility	96 (50.5%)[abcd]	17 (28.8%)[a]	9 (17.3%)[b]	17 (34.0%)[c]	38 (31.1%)[d]	< 0.001
Pain in epigastrium (Cluster 6)	63 (33.2%)[ac]	5 (8.5%)[aef]	16 (30.8%)[e]	7 (14.0%)[c]	37 (30.3%)[f]	0.002
Dysmenorrhea	118 (62.1%)[cbd]	28 (47.5%)	20 (38.5%)[b]	17 (34.0%)[c]	55 (45.1%)[d]	0.002
Dyspareunia	103 (54.2%)[ad]	20 (33.9%)[a]	20 (38.5%)	21 (42.0%)	42 (34.4%)[d]	0.012
Ovulation pain	119 (62.6%)[acd]	27 (45.8%)[a]	23 (44.2%)	21 (42.0%)[c]	58 (47.5%)[d]	0.020
Pain in sternum (Cluster 8)	23 (12.1%)[ac]	1 (1.7%)[a]	5 (9.6%)	0[c]	9 (7.4%)	0.020
Dyschezia	78 (41.1%)[cd]	20 (33.9%)	14 (26.9%)	12 (24.0%)[c]	31 (25.4%)[d]	0.044
Pain in subscapular region (Cluster 11)	51 (26.8%)[ac]	8 (13.6%)[a]	13 (25.0%)	6 (12.0%)[c]	37 (30.3%)	0.044
Pain in right hypochondrium (Cluster 5)	34 (17.9%)	3 (5.1%)	11 (21.2%)	5 (10.0%)	23 (18.9%)	0.078
Pain in pelvis, groin, and sacrum (Cluster 3)	116 (61.1%)	38 (64.4%)	28 (53.8%)	28 (56.0%)	61 (50.0%)	0.407
Abdominal pain for at least 12 weeks	115 (60.5%)	29 (49.2%)	30 (57.7%)	25 (50.0%)	63 (51.6%)	0.460
Pain in urethra (Cluster 2)	13 (6.8%)	8 (13.6%)	2 (3.8%)	3 (6.0%)	11 (9.0%)	0.460
Pain in hips, gluteus, lumbar, upper thigh, vulva, or perineum (Cluster 4)	131 (68.9%)	42 (71.2%)	33 (63.5%)	37 (74.0%)	74 (60.7%)	0.460
Muscle or joint pain	91 (47.9%)	24 (40.7%)	19 (36.5%)	19 (38.0%)	49 (40.2%)	0.509
Pain in lower limbs, upper back, chest, or inner thighs (Cluster 14)	122 (64.2%)	32 (54.2%)	33 (63.5%)	27 (54.0%)	73 (59.8%)	0.560
Dysuria	71 (37.4%)	23 (39.0%)	18 (34.6%)	15 (30.0%)	43 (35.2%)	0.880

This table presents a univariate analysis done with traditional statistical techniques. Significant differences were assessed using Fisher's Exact Test. P-value adjusted for the false discovery rate. Pairwise comparisons for any significant associations were evaluated with Boschloo's Exact Test. [a,b,c,d,e,f] Significantly different pairwise values p-value < 0.05.
