# Supplementary material for: An artificial intelligence approach for investigating multifactorial pain-related features of endometriosis
Source: PLoS One. 2024 Feb 21;19(2):e0297998. doi: 10.1371/journal.pone.0297998 (PMC10881015; doi:10.1371/journal.pone.0297998)
Supplement: S4 Table — For the definition of relative risk (RR), see S4 Fig. Mean estimates with 95% confidence intervals are shown in parentheses. Significant differences were assessed using the independent samples t-test. P-value adjusted for the false discovery rate. * p-value < 0.05; *** p-value < 0.001. (PDF) [file pone.0297998.s004.pdf]

S4 Table. Relative risk of an endometriosis rASRM stage, given a symptom.

**Symptom**	**Early-stage Endometriosis (Stage I & II)**	**Late-stage Endometriosis (Stage III & IV)**
Chronic pelvic pain	2.041 (2.000, 2.081)	2.068 (2.007, 2.129)
Dyspareunia	1.286*** (1.272, 1.300)	1.238*** (1.227, 1.249)
Subfertility	1.285*** (1.268, 1.302)	2.293*** (2.212, 2.374)
Dysmenorrhea	1.252 (1.241, 1.264)	1.256 (1.236, 1.276)
Abdominal pain for at least 12 weeks	1.239 (1.231, 1.247)	1.229 (1.218, 1.239)
Pain in epigastrium (Cluster 6)	1.173* (1.164, 1.182)	1.147* (1.138, 1.157)
Dysuria	1.167* (1.161, 1.173)	1.151* (1.143, 1.159)
Ovulation pain	1.126 (1.118, 1.135)	1.113 (1.106, 1.120)
Dyschezia	1.126 (1.120, 1.132)	1.129 (1.117, 1.142)
Pain in subscapular region (Cluster 11)	1.113* (1.108, 1.118)	1.098* (1.092, 1.104)
Pain in right hypochondrium (Cluster 5)	1.100*** (1.095, 1.105)	1.077*** (1.070, 1.085)
Pain in hips, gluteus, lumbar, upper thigh, vulva, or perineum (Cluster 4)	1.073 (1.069, 1.076)	1.070 (1.066, 1.074)
Pain in lower limbs, upper back, chest, or inner thighs (Cluster 14)	1.073* (1.070, 1.076)	1.065* (1.061, 1.069)
Pain in sternum (Cluster 8)	1.071 (1.066, 1.075)	1.086 (1.069, 1.103)
Muscle or joint pain	1.060* (1.057, 1.062)	1.055* (1.052, 1.058)
Pain in urethra (Cluster 2)	1.034 (1.031, 1.038)	1.043 (1.035, 1.052)
Pain in pelvis, groin, and sacrum (Cluster 3)	1.032 (1.028, 1.036)	1.035 (1.031, 1.038)

For the definition of relative risk (RR), see Fig S4. Mean estimates with 95% confidence intervals are shown in parentheses. Significant differences were assessed using the independent samples t-test. P-value adjusted for the false discovery rate. * p-value < 0.05; *** p-value < 0.001
