# Supplementary material for: An artificial intelligence approach for investigating multifactorial pain-related features of endometriosis
Source: PLoS One. 2024 Feb 21;19(2):e0297998. doi: 10.1371/journal.pone.0297998 (PMC10881015; doi:10.1371/journal.pone.0297998)
Supplement: S5 Table — For the definition of relative risk (RR), see S4 Fig. Mean estimates with 95% confidence intervals are shown in parentheses. Significant differences were assessed using the independent samples t-test. P-value adjusted for the false discovery rate. * p-value < 0.05; *** p-value < 0.001. (PDF) [file pone.0297998.s005.pdf]

S5 Table. Relative risk of an endometriosis typology, given a symptom.

Symptom	Superficial Endometriosis	Deep Infiltrating (DIE) or Endometriomas
Chronic pelvic pain	1.843*** (1.809, 1.877)	2.442*** (2.380, 2.505)
Dyspareunia	1.222*** (1.214, 1.231)	1.299*** (1.289, 1.310)
Dysmenorrhea	1.203*** (1.193, 1.214)	1.272*** (1.259, 1.285)
Subfertility	1.202*** (1.187, 1.216)	2.166*** (2.103, 2.229)
Abdominal pain for at least 12 weeks	1.200*** (1.192, 1.207)	1.272*** (1.262, 1.282)
Pain in epigastrium (Cluster 6)	1.151* (1.142, 1.161)	1.170* (1.161, 1.180)
Dysuria	1.141*** (1.136, 1.147)	1.188*** (1.179, 1.196)
Ovulation pain	1.105*** (1.097, 1.113)	1.131*** (1.124, 1.138)
Dyschezia	1.103*** (1.099, 1.108)	1.150*** (1.138, 1.161)
Pain in subscapular region (Cluster 11)	1.098*** (1.093, 1.103)	1.114*** (1.108, 1.121)
Pain in right hypochondrium (Cluster 5)	1.086 (1.081, 1.092)	1.088 (1.080, 1.095)
Pain in lower limbs, upper back, chest, or inner thighs (Cluster 14)	1.063*** (1.060, 1.066)	1.080*** (1.076, 1.084)
Pain in sternum (Cluster 8)	1.061*** (1.057, 1.066)	1.101*** (1.084, 1.118)
Pain in hips, gluteus, lumbar, upper thigh, vulva, or perineum (Cluster 4)	1.060*** (1.057, 1.063)	1.085*** (1.080, 1.089)
Muscle or joint pain	1.053*** (1.051, 1.056)	1.068*** (1.065, 1.072)
Pain in urethra (Cluster 2)	1.028*** (1.025, 1.032)	1.045*** (1.041, 1.049)
Pain in pelvis, groin, and sacrum (Cluster 3)	1.020*** (1.017, 1.023)	1.041*** (1.034, 1.049)

For the definition of relative risk (RR), see Fig S4. Mean estimates with 95% confidence intervals are shown in parentheses. Significant differences were assessed using the independent samples t-test. P-value adjusted for the false discovery rate. * p-value < 0.05; *** p-value < 0.001
