# Supplementary figures and images for: An artificial intelligence approach for investigating multifactorial pain-related features of endometriosis
Source: PLoS One. 2024 Feb 21;19(2):e0297998. doi: 10.1371/journal.pone.0297998 (PMC10881015; doi:10.1371/journal.pone.0297998)

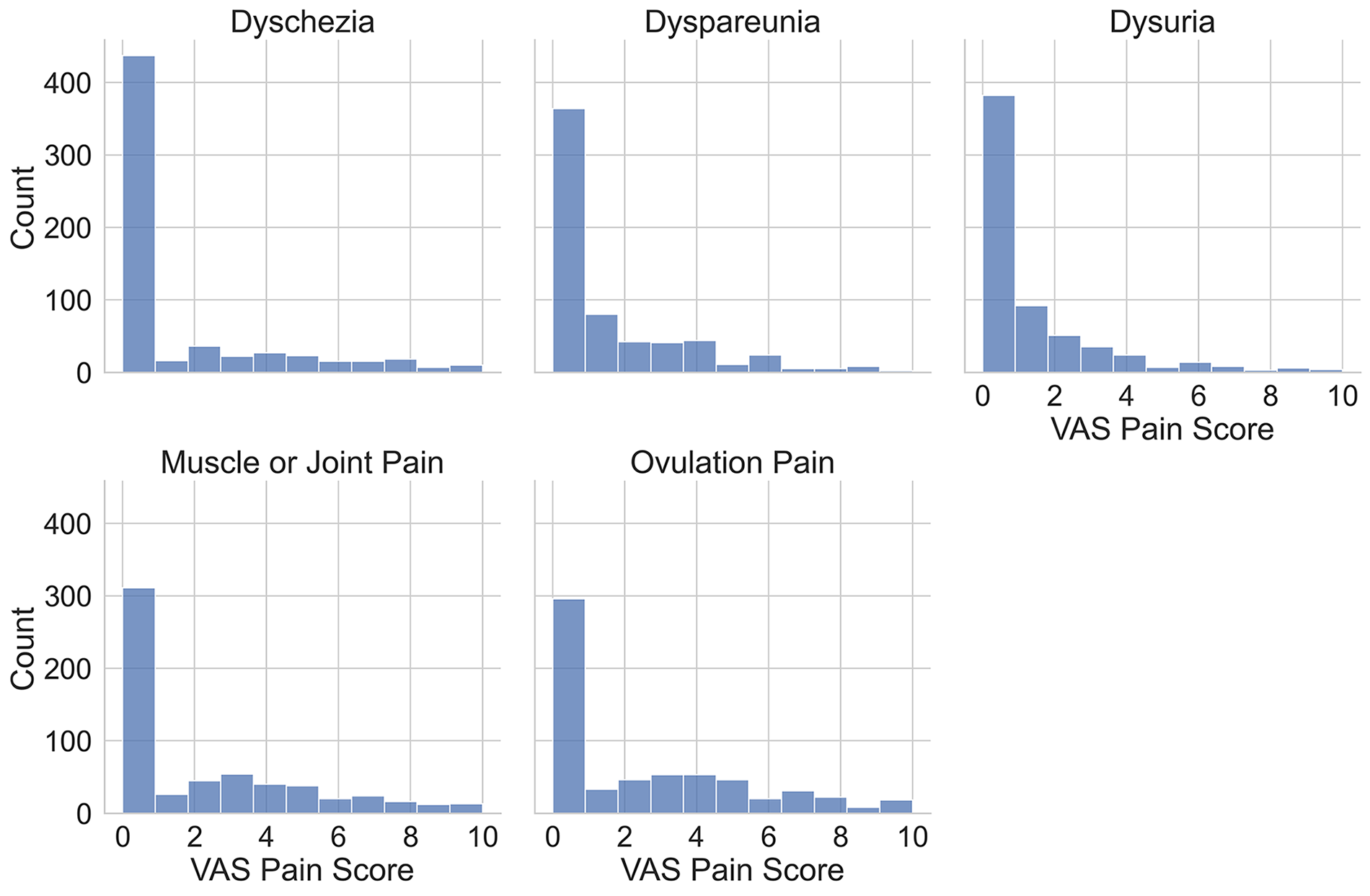

Supplement: S1 Fig — The distribution of VAS pain scores for each pain type. We determined a pain threshold of 1 was most appropriate for dichotomization of pain scores (see S1 Table). (TIF) [file pone.0297998.s006.tif]

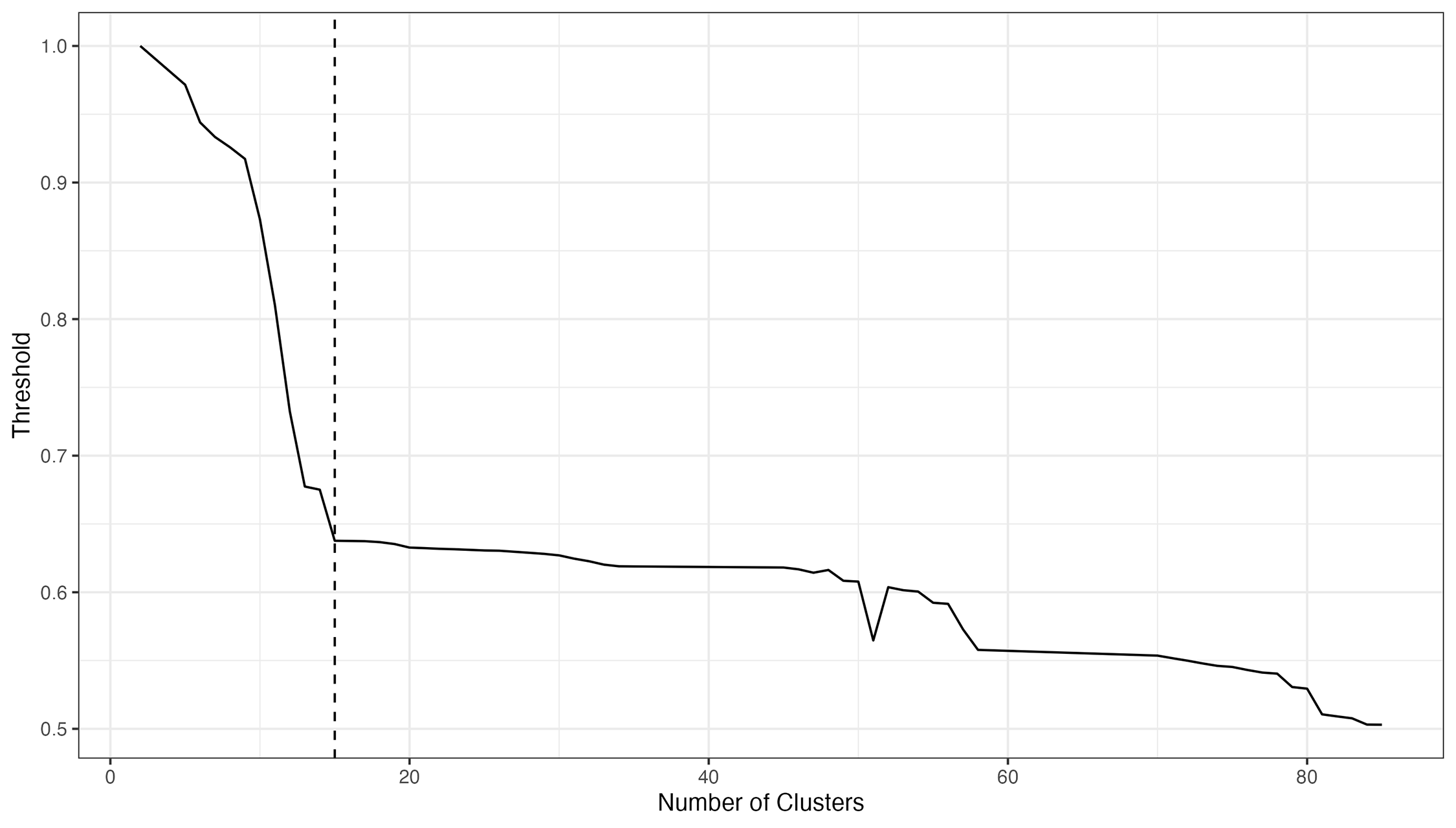

Supplement: S2 Fig — The elbow method requires identifying the point in the chart where a sharp bend, or “elbow,” occurs. This is determined to be the optimal number of clusters that are informative. The threshold represents the maximum distance separating sites within each cluster. In this chart, an elbow can be identified at 15 clusters, corresponding to a threshold of 0.6375. (TIF) [file pone.0297998.s007.tif]

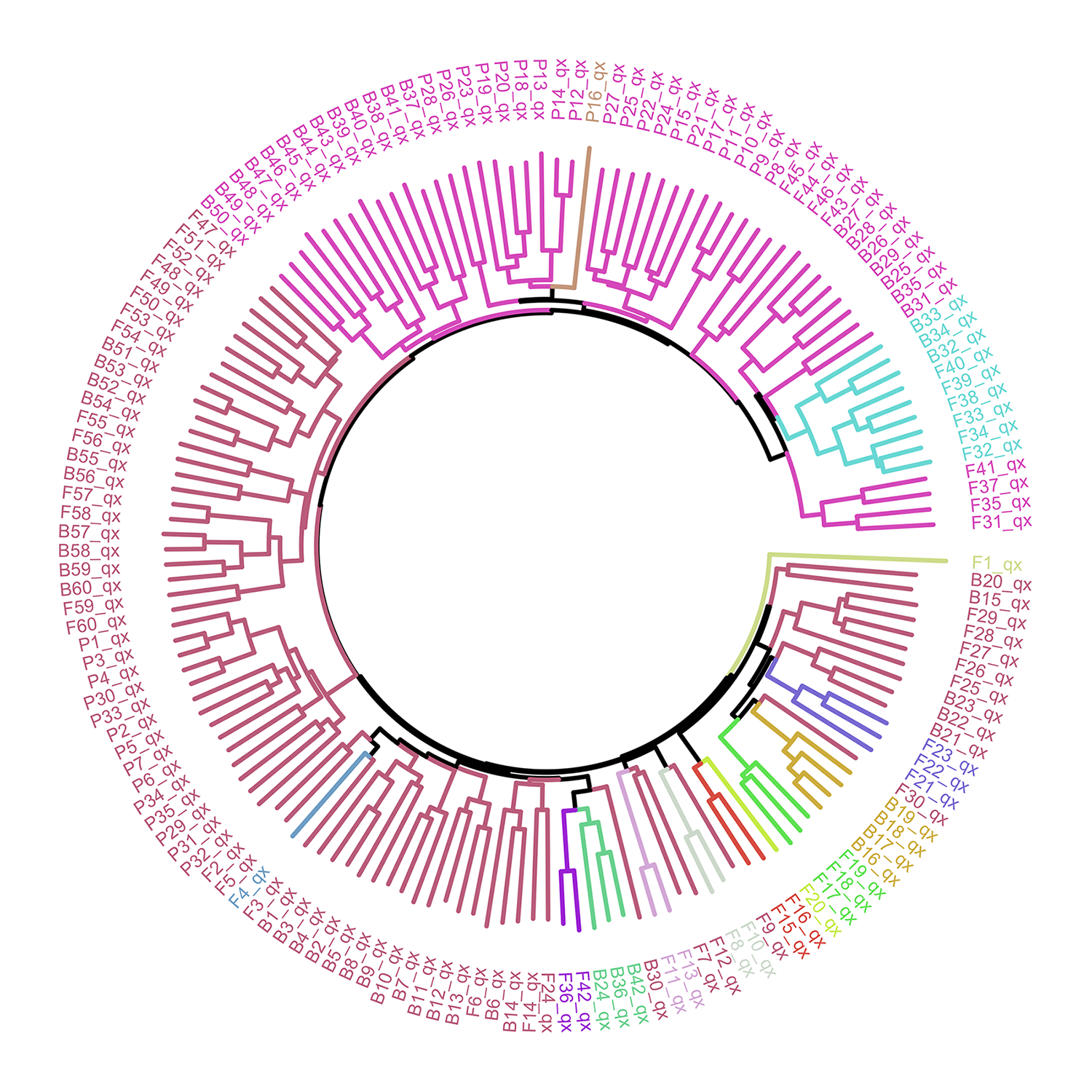

Supplement: S3 Fig — The neighbor-joining algorithm produced an unrooted tree. For visualization as a dendrogram, this tree has been rooted at the midpoint. (TIF) [file pone.0297998.s008.tif]

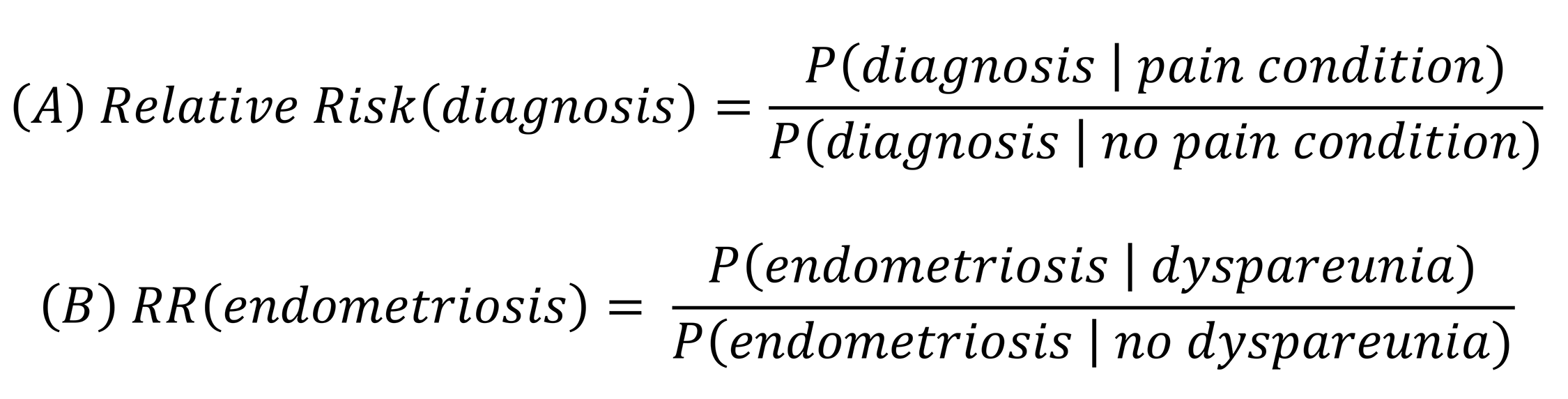

Supplement: S4 Fig — (A) When evaluating the relative risk given a single pain-related feature, relative risk is defined as the absolute risk of having a diagnosis given the presence of a pain type or location compared to the absolute risk of having a diagnosis given the absence of a pain type or location. (B) For example, the relative risk of endometriosis when dyspareunia is present is calculated from the absolute risk of endometriosis when dyspareunia is present divided by the absolute risk of endometriosis when dyspareunia is absent. (TIF) [file pone.0297998.s009.tif]

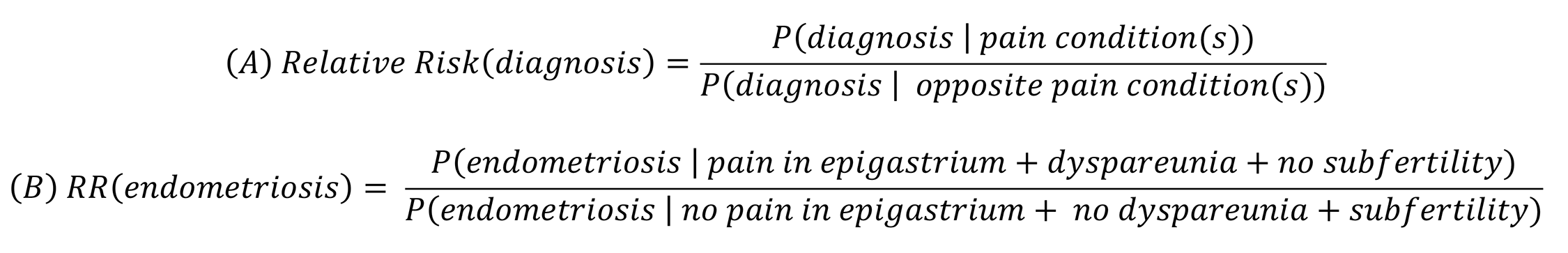

Supplement: S5 Fig — (A) When evaluating the relative risk in the presence or absence of multiple pain-related features, relative risk is defined as the absolute risk of having a diagnosis given the presence or absence of a pain type or location compared to the absolute risk of having a diagnosis given the opposite condition(s). (B) For example, the relative risk of endometriosis when pain in the epigastrium and dyspareunia are present and subfertility is absent is calculated from the absolute risk of endometriosis when pain in the epigastrium and dyspareunia are present and subfertility is absent divided by the absolute risk of endometriosis when pain in the epigastrium and dyspareunia are absent and subfertility is present. (TIF) [file pone.0297998.s010.tif]

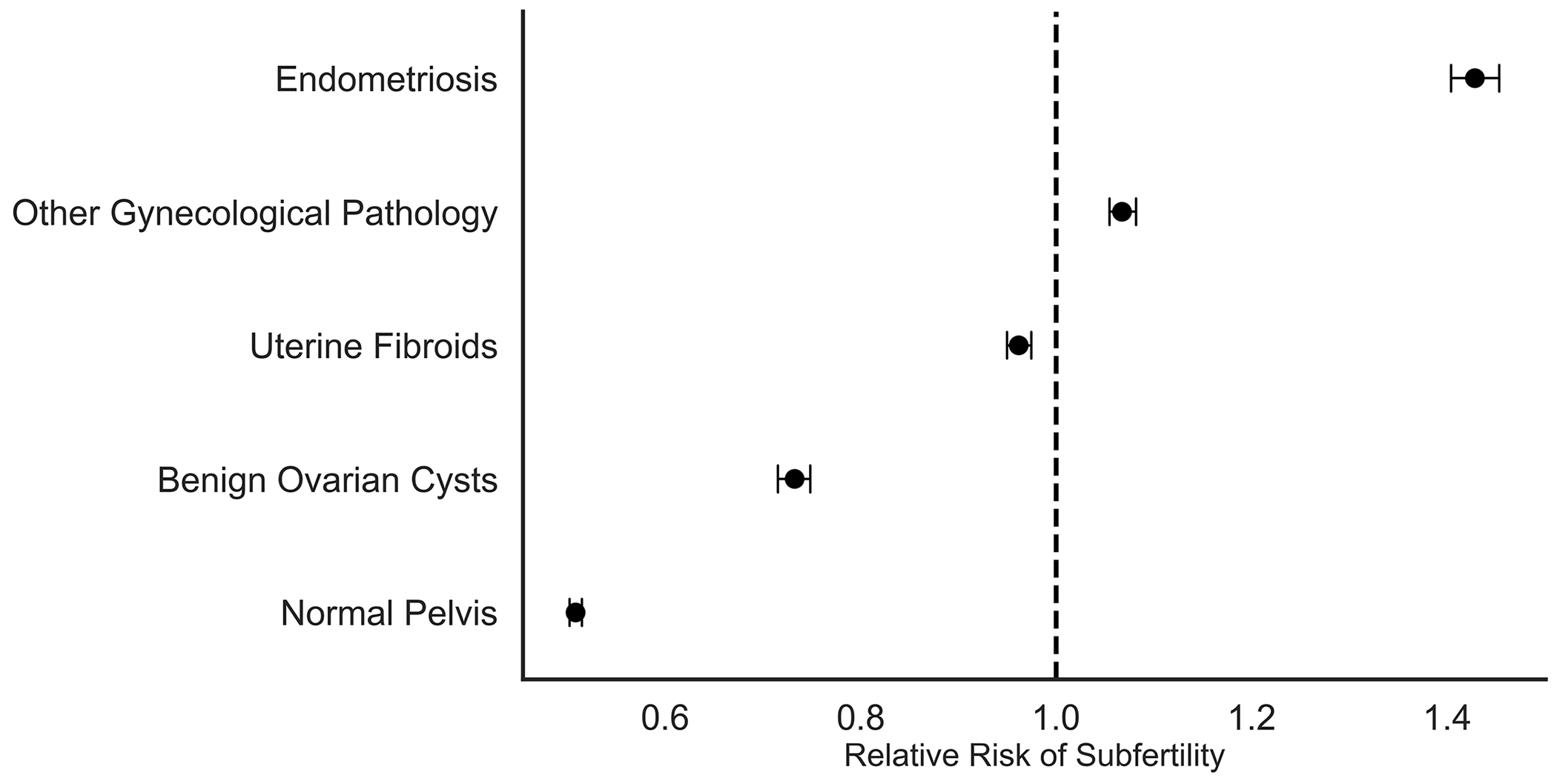

Supplement: S6 Fig — Relative risk of subfertility given a specific diagnosis. This forest plot illustrates the relative risk of subfertility, defined as the absolute risk of subfertility when a specific diagnosis is present compared to the absolute risk of subfertility when a specific diagnosis is absent. Error bars represent the 95% confidence intervals. Values to the right of the dotted line indicate an increased relative risk, whereas values to the left indicate a decreased relative risk. (TIF) [file pone.0297998.s011.tif]

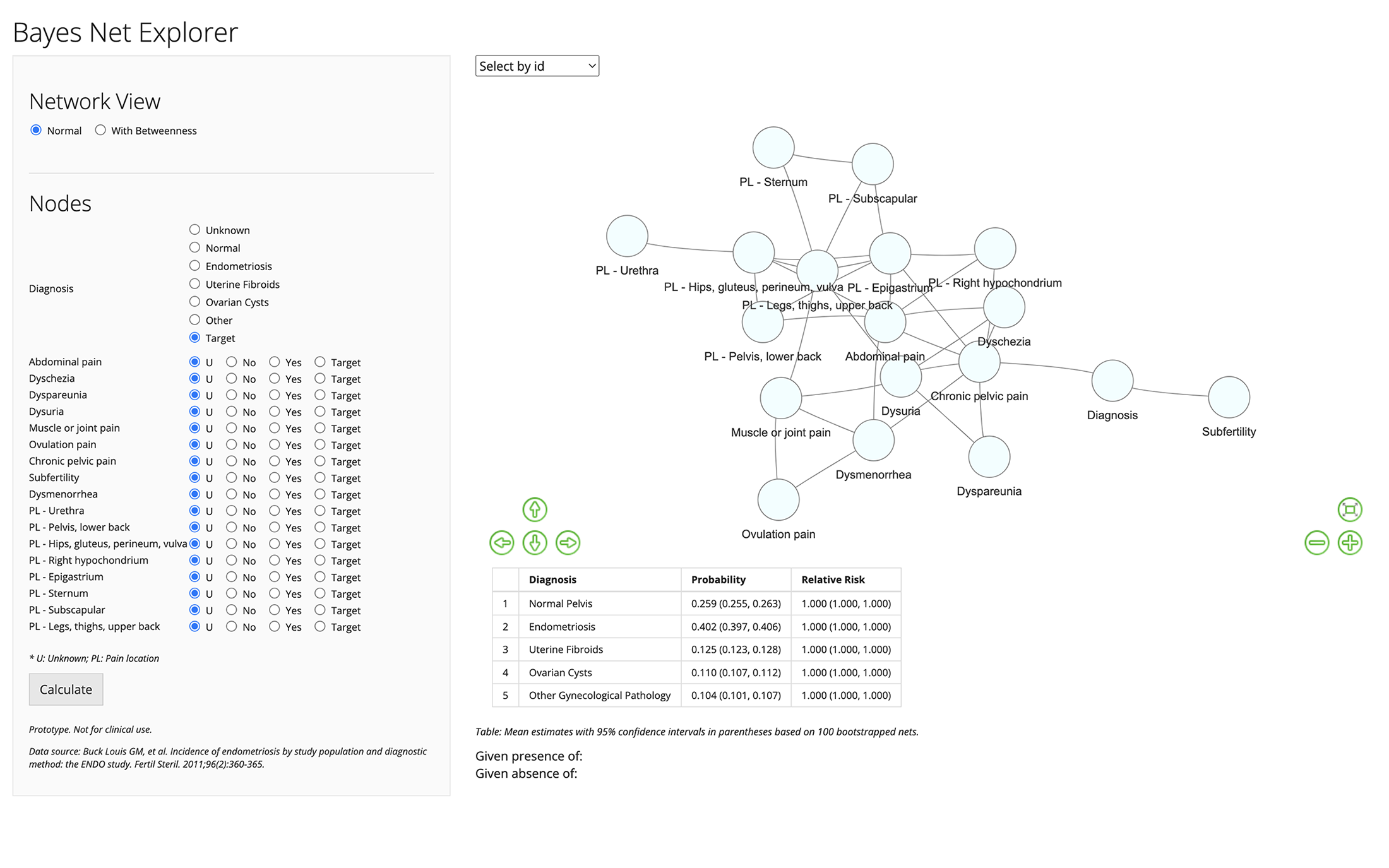

Supplement: S7 Fig — The Bayes Net Explorer app visualizes the calculated Bayesian network and allows users to run any combination of queries, accessible at amber-kiser.shinyapps.io/ENDO-pain-app. (TIF) [file pone.0297998.s012.tif]

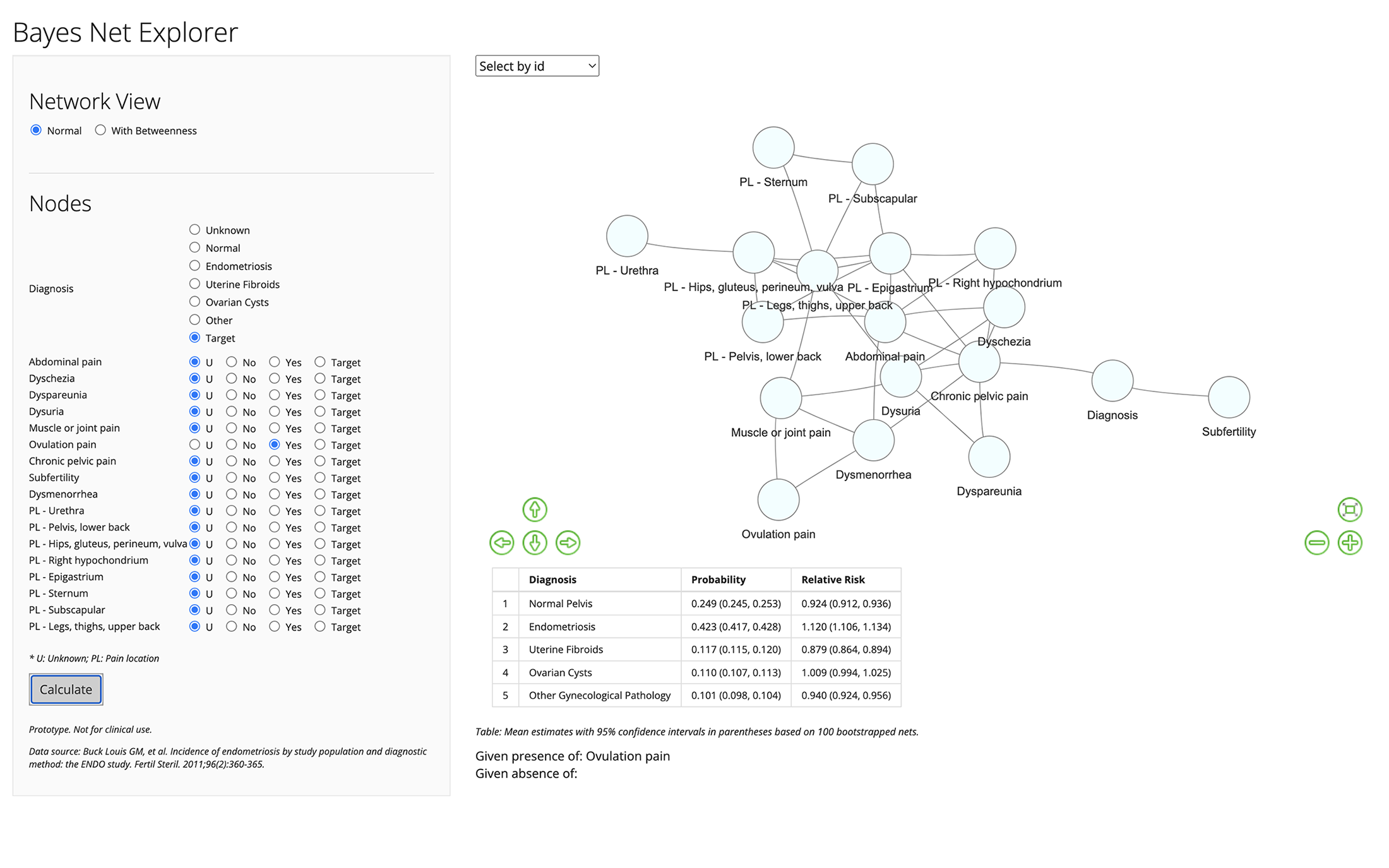

Supplement: S8 Fig — The probability and relative risk of postoperative diagnoses are returned, given the presence of ovulation pain. (TIF) [file pone.0297998.s013.tif]

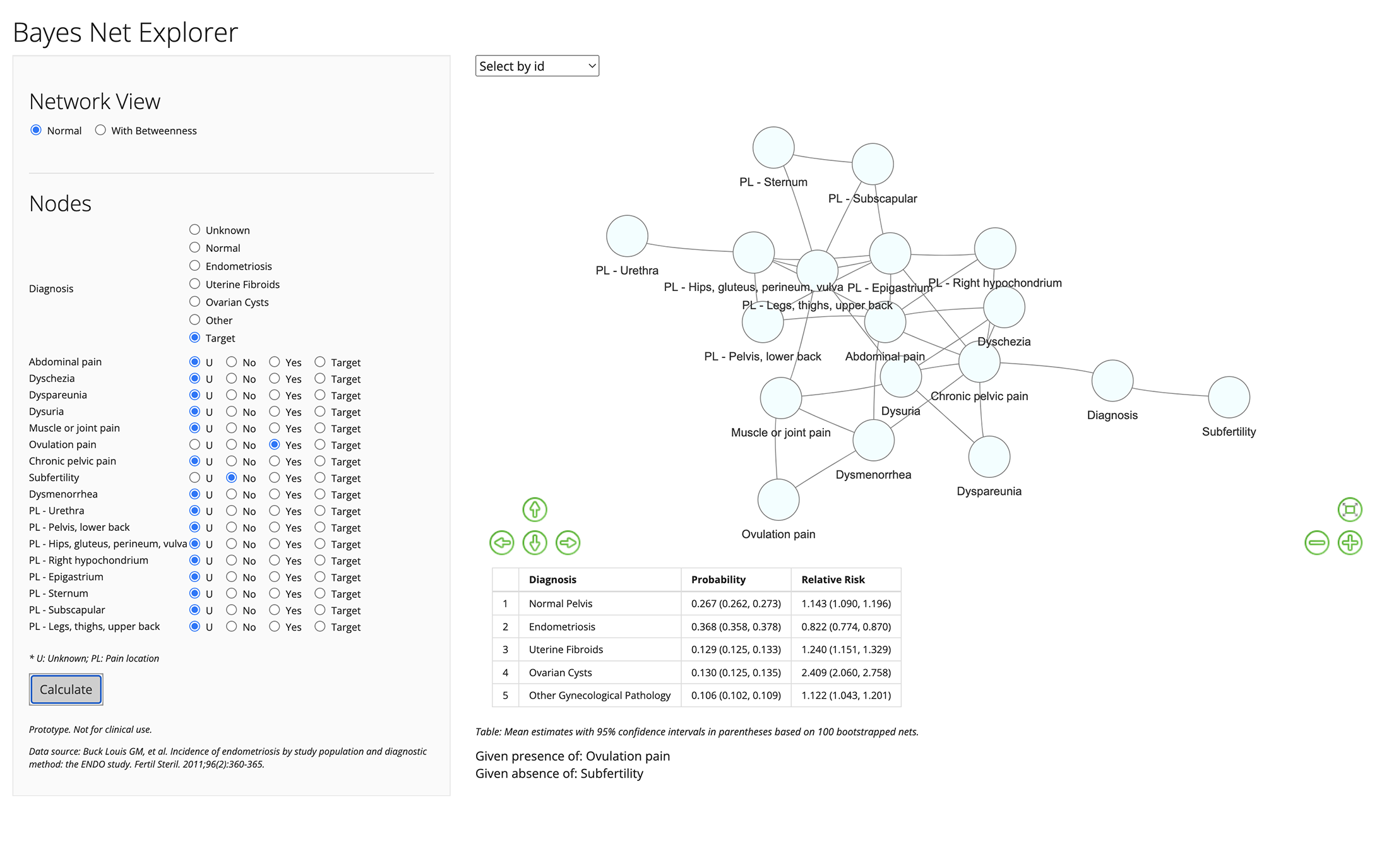

Supplement: S9 Fig — The probability and relative risk of postoperative diagnoses are returned, given the presence of ovulation pain and absence of subfertility. (TIF) [file pone.0297998.s014.tif]

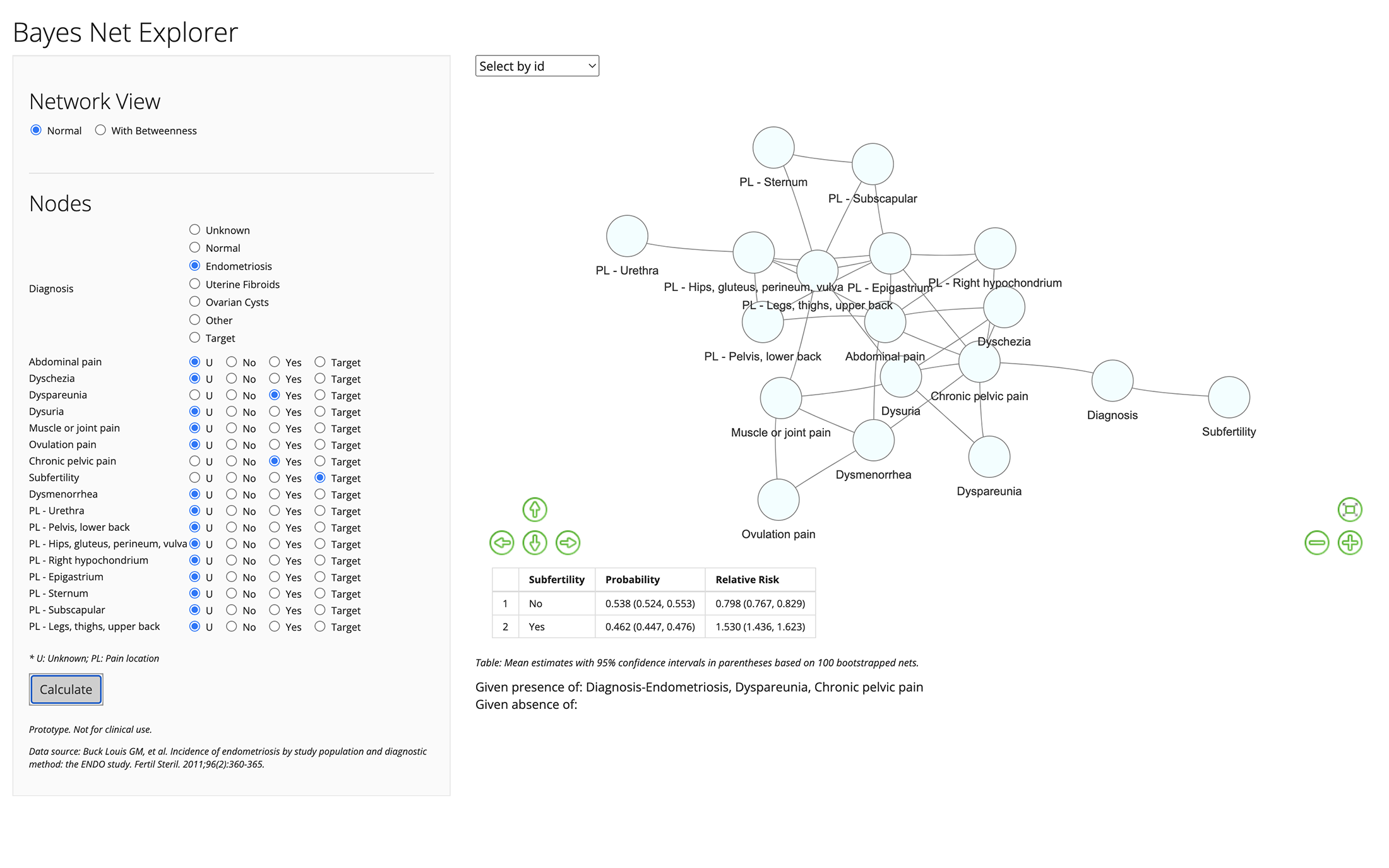

Supplement: S10 Fig — The probability and relative risk of subfertility is returned, given the presence of an endometriosis diagnosis, dyspareunia, and chronic pelvic pain. (TIF) [file pone.0297998.s015.tif]
